# LIVER TRANSPLANTATION AFTER SEVERE HEPATIC TRAUMA: CURRENT INDICATIONS AND RESULTS

**DOI:** 10.1590/S0102-6720201500040017

**Published:** 2015

**Authors:** Marcelo Augusto Fontenelle RIBEIRO-JR, Melina Botelho MEDRADO, Otto Mauro ROSA, Ana Júlia de Deus SILVA, Mariana Prado FONTANA, José CRUVINEL-NETO, Alexandre Zanchenko FONSECA

**Affiliations:** 1 Department of General Surgery of the General Hospital of Grajaú; 2 Santo Amaro University, São Paulo, SP, Brazil

**Keywords:** Liver transplantation, Hepatic trauma, Surgery

## Abstract

***Background*:**

The liver is the most injured organ in abdominal trauma. Currently, the treatment in most cases is non-operative, but surgery may be necessary in severe abdominal trauma with blunt liver damage, especially those that cause uncontrollable bleeding. Despite the damage control approaches in order to achieve hemodynamic stability, many patients develop hypovolemic shock, acute liver failure, multiple organ failure and death. In this context, liver transplantation appears as the lifesaving last resource

***Aim*:**

Analyze the use of liver transplantation as a treatment option for severe liver trauma.

***Methods*:**

Were reviewed 14 articles in the PubMed, Medline and Lilacs databases, selected between 2008-2014 and 10 for this study.

***Results*:**

Were identified 46 cases undergoing liver transplant after liver trauma; the main trauma mechanism was closed/blunt abdominal trauma in 83%, and severe trauma (>grade IV) in 81 %. The transplant can be done, in this context, performing one-stage procedure (damaged organ removed with immediate transplantation), used in 72% of cases. When the two-stage approach is performed, end-to-side temporary portacaval shunt is provided, until new organ becomes available to be transplanted. If two different periods are considered - from 1980 to 2000 and from 2000 to 2014 - the survival rate increased significantly, from 48% to 76%, while the mortality decreased from 52% to 24%.

***Conclusion*:**

Despite with quite restricted indications, liver transplantation in hepatic injury is a therapeutic modality viable and feasible today, and can be used in cases when other therapeutic modalities in short and long term, do not provide the patient survival chances.

## Abstract (pt)

***Introdução*:**

O fígado é um dos órgãos mais afetados nos traumas abdominais. Atualmente, o tratamento de escolha na maioria dos casos é o não operatório; porém, a intervenção cirúrgica pode ser necessária nos traumas abdominais severos com lesão hepática grave, principalmente naqueles que provocam hemorragias de difícil controle. Apesar das abordagens de controle de danos visando a estabilidade hemodinâmica, muitos pacientes evoluem para choque hipovolêmico, insuficiência hepática aguda, falência múltipla de órgãos e óbito. Nesse contexto, o transplante hepático surge como última opção de tratamento.

***Objetivo*:**

Analisar a utilização do transplante hepático como modalidade terapêutica em traumas hepáticos graves.

***Método*:**

Foram revisados 14 artigos obtidos nas bases de dados Pubmed, Medline e Lilacs entre 2008-2014, sendo selecionados 10 para o presente estudo.

***Resultados*:**

Foram identificados 46 relatos de casos de pacientes submetidos à transplante de fígado após trauma hepático; o principal mecanismo de trauma foi o fechado/contuso com 83%, e traumas graves (>grau IV) em 81%. O transplante pôde ser realizado em uma etapa (paciente com órgão lesado removido e imediatamente recebia o enxerto), utilizado em 72% dos casos. Nos procedimentos em duas etapas realizava-se shunt temporário portocava até que um órgão fosse disponibilizado. Na análise de dois períodos distintos - 1980 a 2000 e 2000 a 2014 -, taxa de sobrevida aumentou significativamente, passando de 48% para 76% e a mortalidade caiu de 52% para 24%.

***Conclusão*:**

O transplante hepático apesar de ter indicações bastante restritas no cenário do trauma hepático, representa modalidade terapêutica nos dias de hoje viável e factível, podendo ser empregada em casos onde o tratamento cirúrgico, assim como outras modalidades terapêuticas, não ofereçam ao paciente chances de sobrevida a curto e longo prazo.

## INTRODUCTION

The mortality rate due to liver injuries has fallen significantly in recent years. Surgical cases resulting from these traumas cover only 10% of cases, while 90% are treated conservatively. The main causes of death following severe hepatic trauma are uncontrollable bleeding due to vascular and liver laceration injury and acute liver failure. Both conditions can be treated by selected cases of liver transplantation; however, indications are still well restricted^2^^,^ 7.

In cases of severe liver injury (TSAA grade>IV) mortality rates rise to about 46-80%, and liver transplantation should be considered in cases where all other therapies have failed to achieve hemodynamic stability, making it imperative to adopt damage control measures in order to promote temporary hemostasis until an organ becomes available for transplantation (Table 1)^3^^,^ 7 ^,^ 11 ^,^ 13.

**TABLE 1:** - Classification of liver trauma as proposed by TSAA

**Lesion grade**	**Description**	
I	Hematoma	Subcapsular not expansive, <10% of surface area
	Laceration	Capsular laceration at a bleeding, <1 cm deep parenchymal
II	Hematoma	Subcapsular not expansive, 10-50% surface area: intraparenchymal, non-expansive, <10 cm diameter
	Laceration	Capsular tear, active bleeding: 1-3 cm deep in the parenchyma, <10 cm in length
III	Hematoma	Subcapsular,> 50% of surface area or expansion. Subcapsular hematoma with active bleeding Intraparenchymal hematoma,> 10 cm or expanding
	Laceração	>3 cm in depth
IV	Hematoma	Hematomas roto with active bleeding
	Laceration	Parenchymal disruption involving 25-75% of hepatic lobe or 1-3 segments (Couinaud) in the same lobe
V	Laceration	Parenchymal break involving> 75% of hepatic lobe or> 3 segments (Couinaud) in the same lobe
	Vascular	Fair liver vascular injury (e., vena cava liver retro / main hepatic veins)
VI	Vascular	Liver avulsion

The indications for liver transplantation due to trauma more described in the literature are: uncontrollable continuous bleeding after damage control operation; extensive complex liver lacerations not amenable to surgical correction; extensive lesions of the portal vein, hepatic vein or bile duct that cannot be repaired by surgery; progressive liver failure due to trauma, and hepatic necrosis. In these patients, often the liver transplant is the last therapeutic alternative; however, not all patients are candidates for transplant and that choice should be conducted carefully and individually. Situations such as severe sepsis, multiple organ failure, other serious injuries associated may contraindicate the transplant^2^^,^ 6 ^,^ 7 ^,^ 9 ^,^ 10 ^,^ 14.

There are two types of procedures described in the literature: transplantation in one and two steps. The in one, is the immediate removal of the native liver with subsequent implantation of a new organ. During the procedure performed in two stages there will be a temporary vascular portocaval shunt type to allow the patient to wait for a new body and avoid congestion in mesenteric splanchnic system^2,^ 9 ^,^ 11.

The objective of this study was to analyze the use of liver transplantation as a treatment option for severe liver trauma.

## METHOD

Survey was conducted in the Pubmed, Medline and Lilacs, between 2008-2014 correlating liver trauma and liver transplantation headings. Was found 14 related articles, of which 10 were selected for theme analysis.

## RESULTS

After making systematic literature review of the literature was identified total of 46 case reports of patients undergoing liver transplant after liver trauma; closed/blunt trauma had a higher prevalence with total of 83%, as well as severe trauma (>grade IV, Table 1) with 81% of the votes. The main indication in 52% of cases was the acute liver failure (OR 0.5, CI: 95%, p=0.1941). The technique in one step was the most frequent in 72% of cases. The characteristics of the sample are shown in Table 2.

**TABLE 2:** - Liver transplantation after liver trauma: characteristic of the sample

n = 46		
Gender	Male	24 (52%)
	Female	19 (41%)
	Non specified	3 (7%)
Trauma mechanism	Closed	38 (83%)
	Penetrating	5 (11%)
	Non specified	3 (7%)
Grade	III	4 (8%)
	IV	15 (33%)
	V	19 (41%)
	VI	3 (7%)
	Non specified	5 (11%)
Tecnique	1 step	33 (72%)
	2 steps	13 (28%)
Indications	Acute failure	24(52%)
	Hemorrhage	9 (19%)
	Biliary fistula	2 (5%)
	Secondary biliary cirrhosis	2 (5%)
	Portal vein thrombosis	1 (2%)
	Hepatic necrosis	8 (17%)

The overall survival rate was 63%, and 24% of patients required retransplantation; 65% were transplanted in early to 72 h and the leading cause of postoperative death was 17% of sepsis cases (Table 3).

**TABLE 3:** - Evolution of patients with liver transplant after liver trauma (n=46)

n (%)		
Postoperative survival rate	29 (63%)	
Postoperative mortality rate	17 (37%)	
Retransplants	11 (24%)	
Waiting time to transplant	Precocious (<72 h)	30 (65%)
	Late (>72 h)	16 (35%)
Cause of death	Sepsis	8 (17%)
	PNM	3 (7%)
	Mesenteric ischemia	1 (2%)
	CMV infection	1 (2%)
	Cerebral edema	1 (2%)
	Multiple organ failure	1 (2%)
	Hepatic failure	1 (2%)
	Non specified	1 (2%)

The treatment by means of transplantation has undergone significant improvement in recent years (Table 4). In 88% of cases the major trauma mechanism was the closed/blunt type, as well as severe trauma (>grade IV). The main indication for transplant remains acute liver failure. The bleeding transplant indication was a statistically significant decrease from 33% to 8% (OR: 5.75; 95% CI, p= 0.0365). The technique in one step was the most used in the last decade with a significant increase to 92% of cases, with statistical significance (OR: 0.07; 95% CI p=0.0011). Early transplantation remains a vast majority increased to 68%, and this fact is due to the mastery of technique combined with the good results achieved with transplantation as a treatment modality for chronic diseases and even against patients with acute liver failure. The rate of survival increased significantly, from 48% to 76%, the mortality rate from 52% to 24%, and sepsis remained the main cause of postoperative death, covering half of patients who progressed to death, compared the two periods analyzed.

**TABLE 4:** - Comparative results between two periods analyzed

**Comparing cases**		**1987-2001 (n=21)**	**2002-2014 (n=25)**	**p**
	Male	10 (48%)	11 (44%)	0.5202
	Female	8 (38%)	14 (56%)	0.1803
	Non specified	3 (14%)	0	NA
Trauma mechanism	Closed	16 (76%)	22 (88%)	0.2536
	Penetrating	2 (10%)	3 (12%)	0.5849
	Non specified	3 (14%)	0	NA
Grade	III	1 (5%)	3 (12%)	0.3735
	IV	4 (19%)	11 (44%)	0.0679
	V	9 (43%)	10 (40%)	0.5409
	VI	3 (14%)	0	0.0876
	Non specified	4 (19%)	1 (4%)	NA
Indications	Acute failure	9 (43%)	15 (60%)	0.1941
	Hemorrhage	7 (33%)	2 (8%)	0.0365
	Biliary fistula	2 (10%)	0	0.2028
	Secondary biliary cirrhosis	1 (5%)	1 (4%)	0.7101
	Portal vein thrombosis	0	1 (4%)	0.5434
	Hepatic necrosis	2 (10%)	6 (24%)	0.1853
Technique	1 step	10 (48%)	23 (92%)	0.0011
	2 steps	11 (52%)	2 (8%)	0.0011
Postoperative survival rate		10 (48%)	19 (76%)	0.6065
Postoperative mortality rate		11 (52%)	6 (24%)	0.0462
Retransplants		8 (38%)	3 (12%)	0.0423
Waiting time to transplant	Precocious (<72 h)	13 (62%)	17 (68%)	0.4506
	Late (>72 h)	8 (38%)	8 (32%)	0.4506
Cause of death	Sepsis	5 (45%)	3 (50%)	0.2536
	PNM	2 (18%)	1 (17%)	0.4334
	Mesenteric ischemia	1 (9%)	0	0.4565
	CMV infection	1 (9%)	0	0.4565
	Cerebral edema	0	1 (17%)	0.5434
	Multiple organ failure	0	1 (17%)	0.5434
	Hepatic failure	1 (9%)	0	0.4565
	Non specified	1 (9%)	0	NA

It is observed that the main indication for technical mode in one step was bleeding after damage control with 52%, highlighting the cases of hepatic necrosis in second place with 24%, with statistical significance (OR: undefined CI: 95% p=0.0162). While the main indication, statistically significant for the technique in two steps was acute liver failure with 77% (OR: 0.13; 95% CI p=0.00001, Table 5).

**TABLE 5:** - Indications according to the technical modality adopted

**Indications**	**1 step**	**2 steps**	**TOTAL**	**p**
Hemorrhage	17 (52%)	3 (23%)	20	0.0759
Acute liver failure	3 (9%)	10 (77%)	13	0.0000
Biliary fistula	2 (6%)	0	2	0.5101
Secondary biliary cirrhosis	2 (6%)	0	2	0.5101
Portal vein thrombosis	1 (3%)		1	0.7173
Hepatic necrosis	8 (24%)	0	8	0.0162
TOTAL	33	13	46	

According to the waiting time to transplant the vast majority of both groups was transplanted in early to 72 h (Table 6). The survival rate was higher in undergoing the procedure at a step 70%, while the mortality rate was 46% in patients undergoing therapy in two steps.

**TABLE 6:** - Comparative results according to the technique modality employed

	**1 step (n=33)**	**2 steps (n=13)**	**p**	
Time to transpalant	Precocious (<72 h)	21 (64%)	9 (69%)	0.5003
	Late (>72 h)	12 (36%)	4 (31%)	0.5003
Postoperative survival rate	23 (70%)	7 (54%)	0.2483	
Postoperative mortality rate	10 (30%)	6 (46%)	0,2483	

## DISCUSSION

Mortality rates related to trauma have a strong association with the lethal triad of trauma and the widespread intense inflammatory response. In considering the complexity of trauma patients that may require liver transplantation, which usually are used massive transfusion protocols and mechanical ventilation while waiting for a transplant, justified the worst survival rates in three months when compared to patients transplanted for other indications. However with the advent of greater technical mastery, anesthetic support and care in intensive care, it can be observed reduction in mortality compared to the results obtained in the 80s and 90s when it started the transplants after trauma^9^.

In 1987, it was reported the first case of liver transplant after liver trauma in a patient with vascular and biliary complex injuries after a car accident, using transplantation in one step, which consists of removing the native liver and immediate implementation of the donor liver. In 1988, the transplantation was described in two steps, which is the total hepatectomy followed terminolaterally portocaval shunt, and a second procedure for implementation of the donor liver with the scope to win time while the donor is found. In most studies, patients tolerated up to 36 h without liver after trauma, with reports of a case that remained for 66 h in anhepatic situation. Despite the low survival rate of the procedure into two steps, it was possible to save up to approximately 25% more patients, making more acceptable procedure for acute cases, especially progressive liver failure and uncontrollable hemorrhage^2^^,^ 4 ^,^ 5 ^,^ 9 ^,^ 10 ^,^ 12.

Three different scenarios are described in the statement of transplantation: massive bleeding due to liver damage controllable only with full hepatectomy; liver failure with progressive clinical deterioration in the days following the trauma; and irreparable vascular or biliary injury or evolve secondary to cirrhosis, which is usually later. Different approaches are adopted in accordance with the indication for the procedure. In the first group, in large and uncontrollable bleeding is indicated transplant in two steps with immediate withdrawal of the injured liver that usually leads to hemodynamic instability arising from massive bleeding as a compatible donor is sought, so keeping the patient with temporary portocaval shunt type. In the second group, the progressive failure of the body, can be chosen to perform the transplant by the standard technique, or in the case of severe hemodynamic instability, the technique in two steps, with the intention of faster improvement of symptoms after removal of insufficient liver. In the third scenario the indication is transplant elective procedure by one step, since they are patients with late post-traumatic sequelae as demonstrated in Table 5 10.

The two steps technique is valuable procedure in severe acute cases. Despite the length of anhepatic situation, this type is often the only and last alternative therapy in the hope till a compatible donor is found and the patient has a chance to survive, and get restabilisation during anhepatic after hepatectomy, being observed amazing long-term success rates with survival up to two decades^9^.

It is not surprising that patients undergoing the procedure in two steps have been, in the vast majority (69%), early transplant on within 72 h, since this therapy is recommended in acute cases, as discussed above. Conversely, patients undergoing transplantation in one step, in addition to cover the majority of transplant patients (n=33) had a greater number of late transplantation (n=12), since this is the technique of choice for elective procedures and chronic complications when a compatible donor was previously chosen^10^.

Regarding retransplantation were identified a total of 11 cases in which the main causes were of cholestasis and repeated cholangitis due to ischemic lesions of the bile duct, hepatic artery thrombosis, primary graft failure, and late failure of the transplanted organ by diabetes^2^^,^ 7 ^,^ 10 ^,^ 14.

Current survival after transplantation for liver trauma is about 76% while two decades ago was around 48%. Sepsis is still the main cause of mortality after transplantation (50%), despite a significant drop in the total number of cases over the past 10 years. This increase in survival rates may be attributed to the fact that 20 years ago the signs were smaller and less frequent^10^.

The patients submitted to the standard procedure in one step survival rate is around 70%, while with the two steps is 54%. Such data are justified by the selection of cases where every mode has been indicated. The cases undergoing treatment in two steps usually end up being more severe cases that end up presenting progressive organ failure and therefore have a worse prognosis, increasing with it the mortality^10^.

Another important factor is the transplantation in patients who develop liver necrosis in the days following the trauma; necrotic liver has been seen as toxic agent leading to increased hemodynamic instability. Therefore, total hepatectomy followed by transplantation in two steps is also indicated in these patients. However, the ethical implications of this approach are key, leaving the post-trauma liver transplantation as the only solution, which is, however, entirely dependent on the availability of a adequate donor^9^.

The ethical issue also includes the context of patients received grafts from living donors. Because of the many implications and repercussions that this theme will bring, not being the focus of this study, was identified only one report from a living donor for a patient who received the right lobe of his brother. They were not identified in the review other cases^7^.

With the increase in the number of organ donors, as well as transplants performed in the last decade, today is considered even in cases of trauma such as early as possible approach with the procedure in one step with significant increase in survival^10^.

In this context, liver failure is a challenge that requires urgent liver transplantation, since it leads to worsening of the clinical condition and hemodynamic stability, leading to complications such as cerebral edema and increased intracranial pressure, which can lead to irreversible brain damage to the patient. Thus, was developed an extracorporeal liver support system, that controls these complications, helping to keep patients stable until a suitable donor was found for the viable and available for transplantation. The methods used for extracorporeal support can be classified between biological and non-biological. The bioartificial system uses primary porcine hepatocytes or processed human hepatocytes that are housed within a bioreactor through which blood is pumped into the extracorporeal circuit. The difficulty of finding a compatible donor quickly, coupled with the inability to maintain stable patient in anhepatic stage for long periods of time, they become a major problem in the management of these patients. Thus, it appears as support therapy an alternative, by increasing the survival time where the transplant precocioulycannot be performed^1^^,^ 8.

Despite not having been found in the present review any study that uses this type as the device bridge method for patients who have their organs removed as a result of the trauma extent and therefore were subjected to the use of this type of device, it may have relevant role in increasing survival during anhepatic phase, as well as the waiting time on the list and may allow patients who previouly could not afford to wait for longer times in the list, are likely to be transplanted.

## CONCLUSION

Liver transplantation, despite having very limited information on the stage of hepatic injury, is therapeutic modality viable and feasible on these days and can be used in cases where surgical treatment as well as other therapeutic modalities, do not offer the patient chances of survival to short and long term.
